# Liquid Chromatography with Tandem Mass Spectrometry Analysis of Carboxymethyl Lysine in Indonesian Foods †

**DOI:** 10.3390/molecules29061304

**Published:** 2024-03-15

**Authors:** Patricia Budihartanti Liman, Mulyana, Yenny, Ratna Djuwita

**Affiliations:** 1 Department of Nutrition, Faculty of Medicine, Universitas Trisakti, Jakarta 11440, Indonesia; 2 Nutrition Study Center, Faculty of Medicine, Universitas Trisakti, Jakarta 11440, Indonesia; 3 Department of Clinical Pharmacy, Faculty of Pharmacy, Padjadjaran University, Bandung 45363, Indonesia; arsenicosa10@gmail.com; 4 Department of Pharmacology and Pharmacy, Faculty of Medicine, Universitas Trisakti, Jakarta 11440, Indonesia; yennyfarmako@trisakti.ac.id; 5 Department of Epidemiology, School of Public Health, Universitas Indonesia, Depok 16424, Indonesia; djuwita257@gmail.com

**Keywords:** carboxymethyl lysine, database, food analysis, Indonesian foods, liquid chromatography with tandem mass spectrometry

## Abstract

There is little data on directly measured carboxymethyl lysine (CML) content in Indonesian foods. This study aimed to generate a database of CML values in foods commonly consumed in West Java and West Sumatra. The results were to be used to update our previous estimated CML values. CML values in food samples were measured using high-pressure liquid chromatography with tandem mass spectrometry (HPLC-MS/MS). Food protein content was analyzed by Kjeldahl’s method or inferred from the nutrition facts’ label. A total of 210 food samples were examined, with the food groups of meat and poultry (1.06 mg CML/100 g edible food), and starchy foods (0.21 mg/100 g edible food) having the highest and lowest mean CML levels, respectively. We found that the foods with the top three highest CML content were fried starch dough (*cimol*), fried fish crackers, and chicken *gulai.* The mean of the estimated values (0.80 mg CML/100 g edible food) was higher than the directly measured values (0.66 mg CML/100 g edible food), [*p* < 0.035]. Conclusion: This database provides information on CML values in Indonesian foods, and can be further used to make a guide policy for the selection of foods to reduce non-communicable diseases. Further measurements are needed on Indonesian dishes to complete the database.

## 1. Introduction

Carboxymethyl lysine is one of several advanced glycation end products (AGEs) that is mostly used to measure the AGE content in foods or in the body [1,2,3,4,5] because of its stability and irreversible structure [6,7]. AGEs also known as glycotoxins, are harmful compounds [8,9] that are formed through nonenzymatic reactions between reducing sugars and foods, resulting in a brownish coloration that was first mentioned by Louis Camille Maillard in 1912 [10]. The identification of the CML-producing pathway through degradation of fructoselysine was subsequently described by Ahmed in 1986 [11]. CML is a product of the glycoxidation process through the oxidation of Amadori products or direct reaction of glyoxal with ε-amino lysine groups [12,13].

Circulatory AGEs in the body are associated with the balance of exogenous AGEs from dietary and cigarette smoke, the accumulation of AGEs in the body, endogenous AGEs synthesis, and the clearance of AGEs [7,14]. A correlation between high AGE consumption and circulatory CML was seen in several studies [15,16,17,18,19]; AGE is furthermore believed to be associated with insulin resistance [7,20], obesity [21,22], cardiovascular disease, and renal failure [1,7,23,24,25].

There are two mechanisms of CML-induced disease, namely a receptor-dependent and a receptor-independent pathway. In the receptor-dependent pathway, CML binds to the CML receptor and triggers some cascade reactions and serial signaling pathways [26]. In the receptor-independent pathway, covalent cross-linking reactions of CML with proteins, lipids, or extracellular matrices will occur, resulting in biochemical and cellular impairment [27]. Other models of the mechanism of action of CML were suggested by Chen and Guo, comprising (i) reactive oxygen species formation; (ii) mitochondrial dysfunction; (iii) AGEs acting as antigens in the immune system; and (iv) AGE-induced allergic reaction [5].

AGEs originating from foods contribute more to the AGE pool than endogenous AGEs do [17,28]. In healthy persons, about 10–30% of the amount of CML in the food consumed is absorbed in the intestinal epithelial cell membrane [28,29] as dipeptides by peptidase transporter PEPT1 [30]. The dipeptides are hydrolyzed into amino acids and penetrate into the membrane. CML is usually absorbed via simple diffusion [28,30]. Around 30% of AGEs are excreted in the urine in healthy people, but only 5% in patients with renal failure [10]. The remaining AGEs accumulate in the body [31]. One study has shown that CML consumed from foods in the long term is deposited mostly in the kidneys, colon, ileum, and lungs [5]. Deposits of CML are also found in the brain, skeletal muscle, testis, liver, heart, spleen, and body fat [5,27,32,33]. 

Food preparation has an influence on AGEs’ content through the browning effect and fluorescence formation. Dry-heat cooking techniques such as deep-frying, roasting, baking, and grilling increase the level of AGEs [34] up to 100-fold from that in untreated food [6,35]. High amounts of fat, animal protein, and cereals are known to result in higher AGEs content as compared to vegetables, fruits, and coffee [6,35,36]. Manufactured foods such as processed nuts and canned meat have high AGEs content [37]. Apart from the process of food preparation, it is known that the storage of foods also affects the AGEs content [35].

It is important to have a database of CML in foods if we want to investigate the as-sociation between dietary CML and NCDs. Uribarri et al. in the USA built a large CML database using enzyme-linked immunosorbent assay (ELISA). Hull et al. in the UK and Scheijen et al. in the Netherlands developed CML databases using liquid chroma-tography with tandem mass spectrometry (LC-MS/MS) [36,37,38]. In regard to Asian countries, Takeuchi et al. in Japan investigated various AGEs using ELISA and developed a CML database [39].

We recently developed a CML database of Indonesian foods by estimation from the results of existing studies that used the LC-MS/MS method [40]. The results of the study showed that in the food group of cereals, instant noodles had the highest estimated CML content and were the second largest contributor to CML intake after steamed white rice. Furthermore, we detected that there were twelve paths that involved dietary CML as a mediator on waist circumference (WC) and that most of the paths had a positive association [41]. A one-unit increment in dietary CML was associated with an increase in WC of 0.33 points. The close relationship between AGEs and obesity was also demonstrated through a bibliometric study [23]. This shows the importance of having a CML database of local foods. However, to date, there are no data on the CML content of Indonesian foods in the Indonesian database on food composition, particularly the CML content based on measurement. The primary objective of the present study was to obtain an Indonesian CML database of Indonesian foods using LCMS/MS. The secondary objective was to compare the estimated CML content of Indonesian foods with that measured by LCMS/MS.

## 2. Results

### 2.1. LCMS Optimization and Method Validation

**Figure 1:**
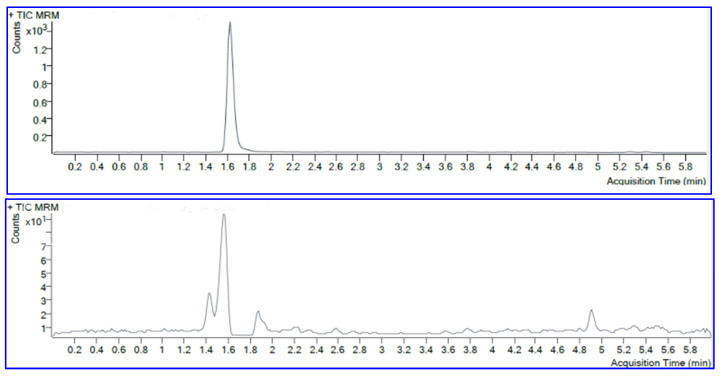
**Upper**: chromatogram of CML standard. **Lower**: chromatogram of CML in fried tilapia.

Although CML is a compound with high polarity, it can still be separated by reversed-phase chromatography. In our study, the analyte was separated with a C18 column, at a column temperature of 40 °C, and gradient mobile phases A and B consisting of 0.1% formic acid in water and 0.1% formic acid in methanol, respectively. Mobile phase A was set up using gradient mode at 0–4 min 80% phase A, and 4–6 min 10% phase A. These conditions gave the most optimal separation results. The resulting LCMS chromatograms of the standard solution (upper chromatogram) and spiked CML in fried tilapia (lower chromatogram) are shown in Figure 1. Optimization of the MRM products was carried out from their highest abundance at *m*/*z* 84.1. The MRM product at *m*/*z* 84.1 was used as a quantification determinant in this study. The MRM internal standard CML-d4 was obtained at *m*/*z* 88.1. The retention times of the CML and CML-d4 standards were measured at the same time. The retention time of the CML separation remained consistent both in the standard solution and in the sample matrix.

#### 2.1.1. Method Validation on Four Types of Food

The calibration equation was y = 3.55 + 3.39x, R^2^ = 0.999, while the linearity range was between 0.5 and 400 μg/L for CML. This is sufficient to determine most CML concentrations in foods. In case of higher CML concentrations in food, the extract can be diluted up to 10 times the targeted level. The limit of detection (LoD) was 0.5 µg/kg in fried tilapia and cornflakes, and 1.5 µg/kg in instant noodles and *pangek sasau*. The limit of quantification (LoQ) was 1.0 µg/kg in fried tilapia and cornflakes, and 5.0 µg/kg in instant noodles and *pangek sasau*. The LoD and LoQ were defined as the concentration (µg/kg) at which the signal-to-noise ratio of the peak of interest was 3 and 10, respectively.

#### 2.1.2. The Recovery of CML-Spiked Samples

The recoveries of exogenous CML-spiked samples were determined at three concentrations, namely at the low concentration of 50 µg/kg, medium concentration of 100 µg/kg and high concentration of 300 µg/kg. The recovery of CML-spiked samples in the sample was almost 100%. The recovery tests were determined at three replications for each concentration. The recovery percentages of each food matrix are shown in Table 1.

The precision test on the samples of fried tilapia, cornflakes, instant noodles, and *pangek sasau* was acceptable. Overall, the precision values obtained from almost all tested matrices are below 10% (Table 2). In the cornflake matrix, the interday precision is slightly above 10%. Sample homogenization is very important in precision and recovery testing.

#### 2.1.3. Quality Testing

##### The Reliability of the Measurement Was Analyzed with Duplicate Measurements 

The reliability of the measurement was analyzed with the incurred sample analysis (ISR) using the same equipment, method, analyst, and laboratory, but on different days. About 15% of the total samples were selected by randomization. In our study, a strong correlation was found between the sample reanalysis of CML content in the foods (y = 0.02 + 0.97x, R = 0.928), with the mean difference between the measurement being 9.9%.

##### Quality Control

**Figure 2:**
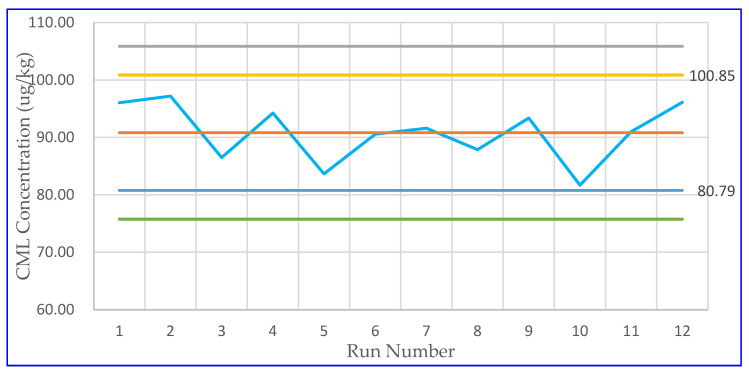
Control chart displaying the variability in the measured concentration of CML.

The quality control in every run day or batch is described in Figure 2. The mean CML concentration derived from 12 quality control food samples is shown by the red line. The yellow and soft blue lines refer to the mean ± 2 SD. The grey and green lines refer to the mean ± 3 SD. The quality control tests using CML spiked in fried tilapia do not exceed the mean ± 2 SD. The QC measurements are closer to the true value.

### 2.2. Measurement of Indonesian Foods

The CML-in-foods database is presented by food group and in units of mg CML/100 g edible food, mg CML/kg protein, and mg CML/average portion, as shown in Table S1. The meat and poultry and starchy food groups have the highest and the lowest mean CML levels with 1.06 mg CML/100 g edible food and 0.21 mg/100 g edible food, respectively (Table 3). When expressed in mg CML/100 g edible food, *kue talam* and *rengginang* have the lowest CML content at 0.01 mg CML/100 g edible food, whereas *cimol* has the highest CML content at 5.35 mg CML/100 g edible food. When expressed in mg CML/kg protein, *ikan maco goreng* has the lowest CML content at 0.37 mg CML/kg protein and *cimol* the highest CML content at 7535.21 mg CML/kg protein.

### 2.3. Estimated versus Directly Measured CML Content

**Figure 3:**
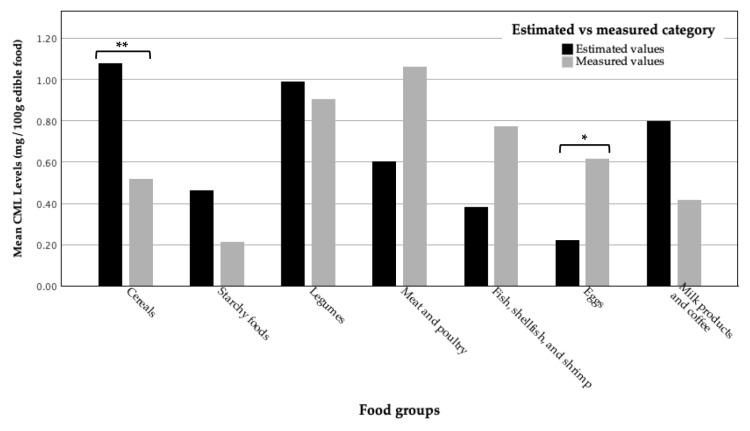
Mean differences between estimated and measured CML content. Wilcoxon test was used to compare continuous data between estimated and measured values of CML content between food groups. * Results were considered statistically significant at *p* < 0.05; and ** at *p* < 0.001.

We found a significant difference between the estimated (0.80 CML/100 g edible food) and directly measured (0.66 CML/100 g edible food) CML values, at *p* = 0.035. When viewed by food group, there was a difference between the estimated and measured CML content in the cereals (*p* < 0.001) and egg groups (*p* = 0.012), but not in the groups of starchy foods, legumes, meat and poultry, fish, shellfish and shrimp, and milk products and coffee (*p* > 0.05 in all groups), as shown in Figure 3.

## 3. Discussion

We measured 210 Indonesian food samples collected in West Sumatra and West Java, Indonesia. As far as we know, ours is the largest CML database with HPLC-MS/MS measurements of Indonesian foods. Foods from these two provinces are the most important focus of discussion about Indonesian foods [42]. One significant difference in the method of food processing between the two provinces that is interesting to explore is the prolonged cooking time by boiling and the dominance of coconut milk in West Sumatran foods as compared to the shorter cooking time by frying and the consumption of fresh vegetable dishes in the foods of West Java [43,44].

The method of CML determination in our study was adopted from the work of He et al. [45]. However, the sample preparation and extraction of CML from foods are different. Food is a sample matrix with a very diverse composition. Food shape, consistency, matrix complexity, protein and fat content, cooking process and analyte content will be challenges in testing CML in food. The processing samples included cutting into smaller pieces, grinding or crushing, defatted process, analyte reduction, hydrolysis, and analyte extraction. In some food samples with high fat content, n-hexane was used for an effective defatting process. Prior to hydrolysis, sodium borohydride was added to reduce the Amadori products (e.g., fructose–lysine) and lipid oxidation products. This step was very important to prevent the formation of CML during acid hydrolysis [46,47].

The cereals group had a higher mean CML/100 g edible food if compared to the starchy group, which is in line with the study of Hull et al. [36]. The mean CML value in the meat and poultry group (1.06 mg CML/100 g edible food) was higher than in the study of Hull et al., who showed that the meat and fish group had a mean CML value of 0.9 mg/100 g edible food [36]. These differences in CML values between the groups may have been due to the differences in food processing, sampling variability, stability of food matrices, and analytical variability.

We found a significant difference between the estimated CML content and the directly measured CML content in the cereal group (*p* < 0.001). The mean estimated CML value in the cereal group was 1.09 mg CML/100 g edible food, which is higher than the mean CML value by direct measurement (0.52 mg CML/100 g edible food). The CML contents of Indonesian sweet snacks in the previous database were estimated with reference to European sweet snacks, which are mostly processed at a high temperature. In contrast, traditional Indonesian snacks such as soft glutinous rice flour cake filled with sweet grated coconut (*bugis*) or coconut cakelets (*bandros*) are prepared at a lower temperature; therefore, the differences in CML values between these food items were large (CML value by estimation for *bugis* and *bandros* was 1.81 mg CML/100 g edible food, and by measurement, for *bugis*, it was 0.67 mg CML/100 g edible food, and for *bandros,* it was 0.14 mg CML/100 g edible food).

Interestingly, the mean CML content of foods in the group of fish, shellfish and shrimp was found to be higher than in Scheijen et al.’s study [37]. The difference could be due to the thickness of fish that was exposed to heat. Chen and Smith [48] measured the CML levels in meat samples, including fried fish fillet of tilapia and salmon, in approximately 2 mm of the outer layer and 2 mm of the middle layer of the fish fillet. Their study showed that all CML values of the outer layer samples were four to sixteen times higher than the CML values of the middle layer. Tilapia fillet had high CML values in the outer layer and even higher values in the middle layer if compared to salmon fillet. The fish samples used in this study were mostly of small-size fish, such as tilapia, *ikan bilis, ikan kembung*, and processed fish, such as salted fish, processed milkfish (*bandeng pindang*), and fish crackers. Salt could increase the CML content through glucose dehydration [35].

The cooking time of the foods apparently increased the CML content during the preparation of beef-based foods. *Gulai*, *kalio*, and *rendang* are the three foods typical of Minangkabau, West Sumatra, that are cooked using identical spices but different cooking times. The first type of Minangkabau food is *gulai*, which is cooked until the total moisture content is reduced and the sauce becomes thin and yellowish in color. The second type is *kalio*, which is cooked for around 1–2 h at a temperature of 90–93 °C, so that the sauce thickens to a brown color. The cooking of *rendang* needs 3–4 h to be complete and sometimes even takes up until 6–7 h at a temperature of 80–93 °C so that the sauce thickens and the meat becomes dry and dark brown in color [43,49]. Our study showed that *gulai* has the lowest CML content in mg CML/100 g edible food, followed by *kalio* and *rendang* with 1.12 mg/100 g, 1.21 mg/100 g, and 1.72 mg/100 g, respectively.

Adding flour into food processing may increase the CML content of food. Cod fish processed by baking had a CML content of about 0.06 mg/100 g, while the CML content in battered cod fish processed by baking increased almost ten times to 0.59 mg/100 g [36]. The increase was also seen in breaded cod fish prepared by baking, which had a CML value of 1.09 mg/100 g. This study also found that fried chicken with added flour had a higher CML content (1.81 mg CML/100 g edible food) than fried chicken without added flour (0.99 mg CML/100 g edible food). Different results were obtained with *tempe mendoan* (lightly fried battered tempeh), which had the same level of CML content (0.63 mg CML/100 g edible food) as *tempe goreng* (tempeh, fried without added flour) with a CML content of 0.67 mg CML/100 g edible food). This may be due to the fact that even though flour is added to the tempeh, *tempe mendoan* has a shorter cooking time than *tempe goreng* (fried tempeh). Besides high heating temperature and low water content, longer cooking time contributes to AGE formation in foods [4,15,20,35].

Protein content has a positive correlation with CML content with r = 0.301 (*p* < 0.001). This is in line with the study of Wu et al., who investigated the influence of shrimp-processing methods on AGE content and showed a positive correlation of protein and oil contents with CML content (*p* < 0.05). Water content had a negative correlation with CML content (*p* < 0.01) [34]. The study of Zhao S et al. [50] also showed a strong positive correlation between protein content and CML content for canned fish with r = 0.46. Differing results were obtained by Fu S. et al. for plant-based food analogs [31] and by Niu L. et al. for commercial fish products [35], both of which did not find a significant correlation between protein and CML content. However, although the correlation between protein and CML content is still subject to controversy, modifications in dietary CML can still be performed without affecting the total protein intakes of the subjects [51,52]. This shows the importance of a CML database as a guideline for selecting foods that are low in AGEs in modifying unhealthy dietary patterns.

Weight per portion has no correlation with CML content, at r = −0.049 (*p* = 0.504). The cereal group, starchy food group, and meat and poultry group that had nearly identical mean weights per portion (79.5 g, 73 g and 64.7 g, respectively) showed very large differences in mean CML contents. The meat and poultry group had the highest mean CML content, namely 1.06 mg CML/100 g edible food, being more than twice the mean CML in the cereal group (0.52 mg CML/100 g edible food), as well as more than five times the mean CML in the group of starchy foods (0.21 mg CML/100 g edible food).

In addition, we examined 14 food items that were taken from the two provinces (Table 4). The mean CML values from West Sumatra and West Java were 0.59 mg/100 g edible food and 0.65 mg/100 g edible food, respectively, at *p*-value 0.290. Although these means were not statistically significantly different, significant differences in CML content were seen in several food items, such as fried chicken breast and boiled noodle.

The CML contents of foods also differed from those in the databases of other countries (Table 5). In our study, the CML content of white rice from West Sumatra was highest at 0.73 mg CML/100 g edible food, whereas that from European countries [37] was lowest at 0.07 mg CML/100 g edible food. White rice samples from West Java had a CML content of 0.24 mg CML/100 g edible food, which was slightly higher than that from the UK [36] at 0.20 mg CML/100 g edible food.

This study recommends the standardization of CML examination procedures that may be used by researchers in the determination of CML levels. Reducing cooking times and refraining from adding flour in the processing of foods may minimize the CML content of these foods. This food database may be used as a reference in estimating CML intakes, and, in turn, may be used to evaluate the relationship between CML intake and disease. Recommendations for the selection of foods may be formulated for the Indonesian communities, particularly those in the two aforementioned provinces.

A limitation of this study is that some food samples were very difficult to homogenize in the preparation process. To date, there is no standard procedure for performing CML measurements; so, each laboratory should carry out CML procedures taken from publications and modify and revalidate these procedures. To our knowledge, this is the first study on CML measurements conducted in Indonesia. Therefore, to minimize bias, the validity and reliability of the measurements were determined with internal standards and CML standards, and performed ISR. One of the objectives of ISR is quality control of the components of measurement to support assay reproducibility. The number of samples to reanalyze for ISR assessments is at least 5% of the study samples in applicable studies. However, we randomly performed an analysis on 15% of all examined samples. The measurements were carried out on different days but in the same laboratory, using the same equipment, method, and analyst. The difference between the measurements was relatively small, 9.9%, which is in accordance with the consensus recommendation that the difference between the concentrations obtained in the initial analysis and the concentrations measured during ISR should be within ±20% [53].

Another limitation is that the food samples were obtained solely from two Indonesian provinces. The present study also did not evaluate in detail the recipes for food processing and the histories of food storage before and after processing, such that these may have resulted in differences in CML content.

## 4. Materials and Methods

### 4.1. Selection of Foods

**Figure 4:**
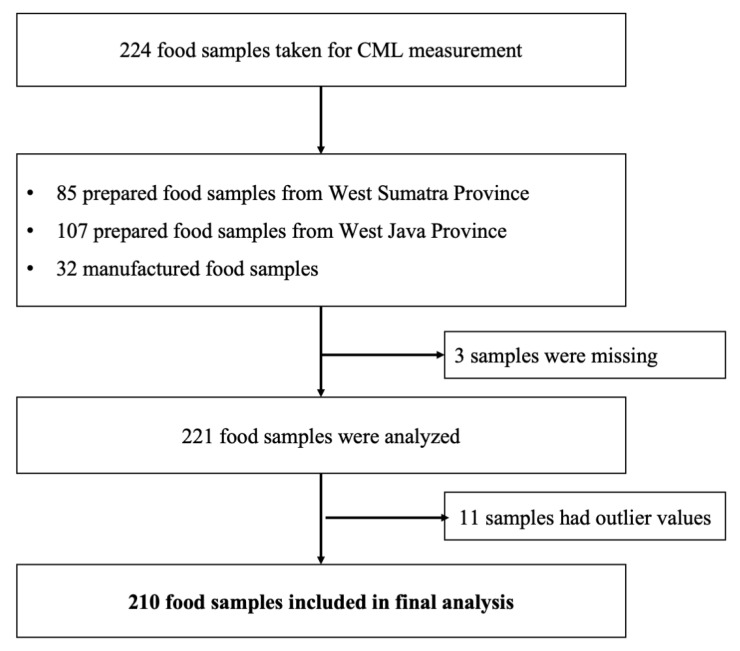
Food sample selection for CML measurement.

Food selection was based on the foods most consumed in the study of Liman et al. [40]. The foods were grouped according to the Indonesian Food Composition Table (TKPI), as described in that study [40]. The food items were listed and ranked according to the highest consumption and included those that were assumed to have a high CML value per 100 g edible food. A total of 224 food samples were selected, consisting of 192 prepared food samples that were obtained from the two provinces and 32 samples of manufactured foods, as shown in Figure 4.

There were three missing food samples and eleven food samples that had extreme values were excluded; so, the total number of food samples included in the final analysis was 210. Average food portions are based on the weight of the food at food sampling or the portion weight stated on the package. Mixed food dishes were examined separately for each food item; for example, chicken porridge was examined separately for rice porridge, shredded boiled chicken, fried cakwe, fried soy beans, and chips. The CML calculation for chicken porridge was based on the CML content of each type of food, the food weight, and the total food weight.

### 4.2. Storage of Samples

Prepared foods were collected from respondents’ homes, stallholders, street food vendors, and from traditional markets. The fast food samples were collected from fast food restaurants, while the manufactured foods were collected from groceries, convenience stores, or minimarkets. Each of the prepared food samples was put in a cooler box provided with Blue Ice packs to prevent oxidation and immediately registered and weighed using calibrated digital scales (Tanita KD-811, Tokyo, Japan). Before handling the samples, the samplers washed their hands with antiseptic soap and handled the foods with clean gloves. Prepared foods were separated into consumable and non-consumable parts using clean spoons and forks, or knives. The non-consumable parts (i.e., bone and sauces) were removed and not measured in this study. The samples were weighed twice to the nearest 0.1 g, each time before and after the non-consumable parts were removed, and the average of those weights was used. Manufactured foods (i.e., cereals, biscuit, chocolate) were kept in their sealed packages. Instant noodles were prepared according to the pack’s instructions at the designated central location. The food samples were placed in dry plastic vacuum containers (Kris vacuum plastic sealer 22 × 500 cm, Hong Kong, China) that did not easily leak. The air in the plastic container was expelled by means of a double-seal vacuum sealer machine (Kris vacuum sealer VS200, China). Each collected food sample was labeled using a permanent marker by stating the sample number, the name of the sample, and the location and time of sampling. The information on the sampler’s name, food type, and cooking method was collected and recorded on a file. The sample was then repacked in a plastic bag to prevent leakage and stored as soon as possible in a freezer at −20 °C until required for measurement. Dry ice was used in transporting the samples from the central location to the laboratory to maintain the temperature.

### 4.3. Determination of CML in Foods

There are four topics that should receive attention in the determination of CML content, namely preparation of samples, instrumentation, LC-MS/MS analytical parameters, and validation.

#### 4.3.1. Preparation of Samples

Before preparing the food samples, the reducing solution was made up. The first step was to make up the borate buffer solution at pH 9.2 and concentration of 400 mmol/L by mixing the first solution consisting of 4.024 g sodium tetraborate in 50 mL ultrapure water with the second solution consisting of 1.2366 g of boric acid in 50 mL ultrapure water, with each solution being stored in a volumetric flask of 50 mL (Pyrex brand, Iwaki, Bandung, Indonesia. To speed up the dissolution process, the mixture was sonicated for five minutes. After both solutions were thoroughly mixed, they were combined and their pH adjusted to 9.2, as determined with S400 digital pH meter (Mettler Toledo, Greifensee, Switzerland), by the addition of NaOH or HCl. After preparing the borate buffer, the sodium borohydrate solution was made up as the reducing solution at a concentration of 100 mmol/L. A quantity of 0.19 g sodium borohydrate was dissolved in 50 mL of the borate buffer solution at pH 9.2.

The next step was the preparation of the food samples to be extracted. The food samples were removed from storage at −20 °C and left to stand at room temperature. Solid food was ground to a paste in a mortar. Then, 100 mg was weighed on scales (Mettler Toledo, type newClassic MF, model MS204S, Columbus, OH, USA, with an accuracy of 0.1 mg–220 mg) and placed in a tube.

Fatty foods with an estimated fat content of more than 20% were defatted in 1 mL n-hexane (Merck KGaA, Darmstadt, Germany, CAS No. 110-54-3). The 20% fat content was obtained from the Indonesian Food Composition Table (TKPI) [54], from Nutrisurvey, from food composition data of ASEAN countries [55], or from the USDA food list [56]. The defatted food mixture was separated by centrifugation (Eppendorf 5702 centrifuge, serial no. 5702BG929940, from Eppendorf AG, 22331 Hamburg, Germany), at 3700 rpm for 5 min, and the precipitate was collected for CML extraction.

The sample was then reduced with the addition of 1 mL of reducing solution, as prepared previously, and left to stand for two hours at a temperature at 23–25 °C (room temperature). After that, 1 mL HCl 6N/12% was added to the sample in the reducing solution; the mixture was then incubated for 30 min in a hot digestion system (ROCKER, Rocker Scientific Co., Ltd., COD reactor, model CR25, cat. no. 179200-22, Taiwan, China) at 110 °C. The purpose of the COD reaction is to determine the amount of organic matter, in this case, originating from the fat content of the food.

After incubation, 990 μL methanol (Tedia Company Inc, Fairfield, OH, USA, lot no. 18080199) and 10 μL internal standard (iSTD) comprising Nε-(1-Carboxymethyl)-L-Lysine-(4,4,5,5-d4) from Cambridge Isotope Laboratories, Inc., Tewksbury, MA, USA, were added. The solution was then sonicated (Bransonic 3510E-DTH, ultrasonic cleaner, Branson Ultrasonics Corporation, Danbury, CT, USA, made in Mexico) for 10–20 min at 23–25 °C, and homogenized by vortexing (approximately 10–20 s). The sample mixture was separated by centrifugation at 3700 rpm for 10 min. Then, 500 μL of the supernatant was taken with a micropipette and inserted into the HPLC vial, at which the samples are ready to be analyzed with HPLC-MS/MS.

The CML contents in the foods were calculated with the following formula:

$$CML\;content\;in\;food(mg/kg=\frac{c\cdot df\cdot v}{W})$$

where the following definitions apply:

c: CML content from LC MS/MS detection;
df: dilution factor;
v: sample volume (L);
W: sample weight (kg).

The expression of CML in food as CML mg/100 g edible food was calculated by the formula:

$$CML\;content\;(mg/100\;g\;edible\;food)=\frac{CML\;content\;in\;food\;(mg/kg)}{10}$$

The expression of CML in food as CML mg/kg protein was calculated by the following formula:

$$CML\;content\;(mg/kg\;protein)=\frac{CML\;content\left(mg/100\;g\;edible\;food\right)\cdot 1000}{Protein\;content\;in\;food\;(g/100\;g\;edible\;food)}$$

Similarly, the expression of CML in food as CML mg/average portion size was calculated by the formula:

$$CML\;content\;(mg/average\;portion)=\frac{CML\;content\;(mg/100\;g\;edible\;food)\cdot portion\;weight\;(g)}{100}$$

#### 4.3.2. Instrumentation

The LC method was carried out on an Agilent 1260 Infinity II system (Santa Clara, CA, USA), Agilent SB-C18 column with dimension of 2.1 × 50 mm, 1.7 μm (Agilent Technologies, CA, USA), and Agilent Ultivo Triple Quadrupole Mass Spectrometer (Santa Clara, CA, USA).

#### 4.3.3. LC-MS/MS Analytical Parameters

A 10 μL aliquot of sample extract was injected into the HPLC MS/MS system at a column temperature of 40 °C. The composition of the mobile phase was 0.1% formic acid in water (mobile phase A), and 0.1% formic acid in methanol (mobile phase B). The mobile phase was set up using gradient mode at 0–4 min 80% phase A and 4–6 min 10% phase A. The LC setting for accurate and reliable results was developed with a 1260 Infinity II system (Santa Clara, CA, USA), using Agilent Jet Stream (AJS) Positive ESI mode as ion source. The drying gas temperature was 350 °C with a gas flow of 8 L/minute, nebulizer pressure was 35 psi with sheath gas temperature of 350 °C and gas flow of 8 L/minute. Capillary voltage, nozzle voltage, and delta EMV were 3000 V, 0 V, and 500 V, respectively. The target analyte structure was Nε-carboxymethyl lysine. Multiple Reaction Monitoring (MRM) transition parameters for the analytes are described in Table 6.

#### 4.3.4. Method Validation

CML contents in foods were determined in the Prodia Industrial Toxicology Laboratory, Cikarang, West Java, Indonesia. This method protocol was validated for selected foods, including fried tilapia, *ikan pangek sasau* (processed traditional Minangkabau fish), cornflakes, and fried instant noodles.

Fried tilapia, cornflakes, instant noodles, and *pangek sasau* were chosen to represent a variety of food types. Fried tilapia and *ikan pangek sasau* represent foods rich in protein and fat but low in carbohydrates. The difference between these two foods lies in the processing method. Fried tilapia is processed by frying in hot oil, but *ikan pangek sasau* is processed by being steamed with spices and coconut milk. Cornflakes and instant noodles represent carbohydrate-rich foods and are processed at high temperature. Cornflakes are served straight away without any further processing. Instant noodles are processed through boiling with the addition of oil, soy sauce, and spices. For the selection of food matrices, the CML content and cooking process should be taken into consideration.

##### Optimization of LCMS/MS Conditions

CML is a polar agent and is difficult to separate in a non-polar column. The analytical method was adopted from the work of He et al. [45]. The C18 column (Agilent Zorbax Eclipse plus C18; 4.6 × 10 mm, 5 μm) was evaluated for the separation of CML. The solutions of formic acid in water and formic acid in methanol were used as mobile phase at a flow rate of 0.5 mL/minute with gradient condition. The column temperature was set at 40 °C. The chosen mobile phases in gradient mode consisting of 0–4 min 80% phase A and 4–6 min 10% phase B showed a better chromatogram. MRM optimization was conducted using MassHunter software v.1.1 for Ultivo LC/TQ C.01.00 2018.

##### Preparation of Calibration Standard and Linearity of the Calibration Curve

The stock solution was made by solubilizing 1 mg of CML and CML-d4 in water, aliquoting and storing at −20 °C. The stock solutions of CML were freshly diluted using mobile phase A concentrations 0.5, 2.5, 5, 10, 25, 100, 200 and 400 μg/L. The internal standard CML-d4 was prepared in the same way at a concentration of 50 μg/L. The calibration curves were determined in duplicate on three consecutive days and their linearity was evaluated. The intensity ratio of CML to CML-d4 and the concentration of CML were determined to make a standard curve. The deviation of the calculated concentrations was within ±15% of the nominal concentration. The limit of quantification was determined with six replicates.

##### Precision

Intraday and interday precision were determined by testing three replicates of three levels in three consecutive days. It is expressed as a percentage and is obtained by multiplying the standard deviation by 100 and dividing this product by the average. The precision was described as a percentage of relative standard deviation (% RSD). The %RSD was acceptable within ±15% of the nominal values.

##### Extraction Recovery

Recovery was evaluated by comparing and spiking the food samples with CML standard (50, 100 and 300 mg/kg). The extraction recovery was determined with the following formula:

$$Recovery\;(\%)=\frac{C_{2}-C_{1}}{C}$$

C: level of CML CML spiking;

C_1_: level of CML in food;

C_2_: level of CML spiking and CML in food.

Limit of Detection and Limit of Quantification (LoD and LoQ)

The LoD and LoQ were determined by the signal-to-noise ratio of the peak of the analytical target at 3 and 10, respectively. LoD and LoQ are expressed in ug/kg.

#### 4.3.5. Quality Testing

##### Reliability of Measurement

The reliability of the measurement was analyzed with the sample reanalysis of 15% of the total samples of the prepared and manufactured foods that were selected by randomization.

##### Quality Control

Fried tilapia was used as a sample base for quality control. Fried tilapia was obtained and selected around 200 g of fish meat. The fish meat was homogenized with a chopper, and the CML content was measured. The CML content of fish meat was used as the baseline CML value. Some of the fish meat was taken to be used in sample-based QC. The CML solution was added with a concentration of about 1 mg/kg and homogenized until it was a porridge-like mass. The mixture should produce a final spiked concentration of 100 µg/kg. The slurry mass obtained was 50 g, which was then aliquoted into plastic containers of 1 g each for QC testing. Before being used, these QC samples were stored in the freezer at −20 °C. Sample-based QC was taken every running day. Storage, preparation, and extraction up to CML content analysis were carried out by the same method as the test sample. QC sample testing was carried out in the middle of the sample testing series. In 1 running day, around 20 samples were analyzed for their CML content. The results of testing of the QC samples were calculated and documented using a control chart.

### 4.4. Determination of Protein Content

Protein levels in foods were determined in the other accredited and standardized laboratory in West Java, Indonesia. The protein content from prepared foods was measured by the Kjeldahl method, which refers to the standard procedure SNI 01-2891-1992, point 7.1. in the Indonesian national standards guideline on food and drinks testing. The protein content of manufactured foods was read from the nutrition facts’ label on the packaging or measured by the Kjeldahl method if there was no label. Briefly, 1 g sample (for high protein content 0.3–0.5 g of sample was used) was put in the Kjeltec tube, and 1 g selenium and 12 mL concentrated H_2_SO_4_ were added. The homogenate was then heated in a block digester (Kjel Digester K-446, Buchi Labortechnik AG, Flawil, Switzerland) at 420 °C for 2 h. Then, the Kjel Digester was turned off and the homogenate removed and left to cool at room temperature. After cooling, 3 drops of phenolphthalein (PP), 50 mL 40% NaOH, and 25 mL distilled water were added. Distillation in a steam distillation system (Buchi distillation K-355, Buchi Labortechnik AG, Switzerland) was then carried out for around 10 min, using 4% boric acid as the absorbing solution to three times its initial volume of 50 mL. The distillate was titrated with a solution of 0.2 N HCl to a red endpoint. Then, the blanks were titrated (Kjeltec system 2020 digestor, Tecator Inc., Herndon, VA, USA).

The following formula was used to calculate the protein content:

$$Protein\;content\;(\%)=\frac{(Vs-Vb)\times N\times 1.4007\times fk}{m\;(grams)}$$

where Vs = volume of sample, Vb = volume of blank, N = normality of titrating solution, m = sample weight, and fk = species-specific nitrogen-to-protein conversion factor, with the following values: food in general = 6.25, milk and dairy products = 6.38, butter and nuts = 5.46, UHT milk = 7.0, peanuts = 5.46, soybeans = 5.71, coconut = 5.30, wheat = 5.38, rice = 5.95.

### 4.5. Statistical Analysis

SPSS program version 28.0.1.1. was used for analyzing the data, with the following details: the Kolmogorov–Smirnov test was used for testing the normality of the data, while the Wilcoxon test was used to determine the correlation between the estimated and measured CML content, the Mann–Whitney test was used to analyze the between-group differences in CML content from the two provinces, and Spearman’s rank correlation test was used to analyze correlation between protein content and weight per portion with CML content. The significance level was set at *p* < 0.05.

## 5. Conclusions

We present our CML database of Indonesian foods, which can be further used to make a guide policy for the selection of foods and their processing or for designing intervention studies with restricted CML intake to reduce non-communicable disease complications. The mean of the estimated values was statistically higher than that of the directly measured values. Therefore, future studies are required to measure the CML content of a larger number of food items from different locations with specific food-processing procedures to complete the database.

## Figures and Tables

**Table 1:** The recovery percentages of CML spiked in fried tilapia, cornflakes, instant noodles and *pangek sasau*.

Food Matrices	Recovery (%)		
	50 (µg/kg)	100 (µg/kg)	300 (µg/kg)
Fried tilapia	86.50–97.20	83.66–94.24	87.87–93.37
Cornflakes	95.52–97.58	101.30–111.55	103.43–106.27
Instant noodles	106.95–110.05	100.54–105.81	81.70–96.11
*Pangek sasau*	98.94–108.14	90.24–102.95	102.62–104.77

**Table 2:** The precision percentage of CML spiked in fried tilapia, cornflakes, instant noodles, and *pangek sasau*.

Food Matrices	Precision (%)	
	Intraday	Interday
Fried tilapia	0.49–0.56	3.09–6.0
Cornflakes	0.63–2.40	4.17–12.83
Instant noodles	1.10–8.16	2.70–8.16
*Pangek sasau*	1.08–4.54	2.88–5.97

**Table 3:** Mean and range of the CML content of food groups, expressed per mg/100 g edible food, mg/kg protein, and mg/average portion size.

Food Groups		mg CML/100 g Edible Food	mg CML/kg Protein	mg CML/Average Portion Size
Cereals	Mean	0.52	209.96	0.36
	Range	0.01–5.35	1.00–7535.21	<0.01–5.29
	Standard deviation	0.83	878.23	0.76
Starchy foods	Mean	0.21	113.71	0.14
	Range	0.06–0.82	31.25–220.00	0.01–0.58
	Standard deviation	0.22	59.56	0.15
Legumes	Mean	0.9	98.59	0.38
	Range	0.02–4.30	1.69–477.40	0.01–1.45
	Standard deviation	0.97	129.50	0.39
Meat and poultry	Mean	1.06	57.05	0.75
	Range	0.04–4.41	4.32–229.58	0.01–3.40
	Standard deviation	1.02	62.54	0.95
Fish, shellfish, and shrimp	Mean	0.77	41.41	0.32
	Range	0.02–5.19	0.37–281.45	0.01–2.34
	Standard deviation	1.05	61.33	0.49
Eggs	Mean	0.61	39.88	0.19
	Range	0.04–2.10	3.17–108.81	0.02–0.53
	Standard deviation	0.56	31.04	0.16
Milk products and coffee	Mean	0.42	73.54	0.15
	Range	0.13–0.95	7.82–161.02	0.01–0.46
	Standard deviation	0.36	63.07	0.17

**Table 4:** Comparison of CML content of foods from two provinces.

Food Name, English	Food Name, Indonesian	West Java (mg CML/100 g Edible Food)	West Sumatra (mg CML/100 g Edible Food)
Chicken, meat, breast, boiled	Ayam, dada, rebus	0.11	0.18
Chicken, meat, breast, fried	Ayam, dada, goreng	0.37	2.25
Chicken, meat, breast, grilled	Ayam, dada, bakar	0.7	1.33
Chips, cassava, home made	Keripik singkong, produk rumahan	0.02	0.09
Meat balls, boiled	Bakso polos, daging sapi, rebus	1.05	1.94
Noodle, boiled	Mi basah	4.15	0.37
Omelet	Telur ayam, dadar	0.5	0.52
Peanut sauce	Bumbu kacang	0.17	0.19
Rice cake boiled in a rhombus-shaped packet of plaited young coconut leaves	Ketupat	0.13	0.28
Tapioca crackers, grilled	Opak bakar	0.05	0.11
Tempeh, fried	Tempe goreng	0.56	0.79
Vegetable fritters	Bala-bala/bakwan	0.04	0.1
White rice, cooked	Nasi putih	0.24	0.73
Noodle, yellow, boiled	Mi kuning rebus	0.17	0.28

**Table 5:** CML content of Indonesian foods compared with European and UK food databases.

Food Name, English	Food Name, Indonesian	West Java	West Sumatra	UK [36]	European [37]
Chicken, meat, breast, boiled	Ayam, dada, rebus	0.11	0.18	0.38	0.18
Chicken, meat, breast, fried	Ayam, dada, goreng	0.37	2.25	0.51	0.34
Cornflakes	Corn flakes	1.19	-	3.47	0.66
Egg noodles	Mi telur	0.19	-	0.30	-
Egg, chicken, fried	Telur ayam goreng	-	0.84	0.63	0.42
Fried rice with egg	Nasi goreng telur	0.84	-	0.09	0.96
Meatballs,	Bakso	1.05	1.94	-	0.83
Omelet	Telur dadar	0.5	0.52	0.78	-
Chocolate milk	Coklat	0.79	-	-	0.96
Tofu, fried	Tahu goreng	1.13	-	-	0.94
Ultra-high-temperature pasteurized milk	Susu UHT	0.23	-	0.22	-
White bread	Roti tawar	-	0.52	0.66	0.24
White rice, cooked	Nasi putih	0.24	0.73	0.20	0.07

**Table 6:** Multiple Reaction Monitoring parameters of CML.

Compound	Precursor Ion (*m*/*z*)	Product Ion (*m*/*z*)	Fragmentor (V)	CE (V)	CAV (V)	Dwell (ms)
CML-d4	209.1	88.1	108	13	9	200
CML	205.1	84.1	94	13	9	200

Abbreviation: CML: carboxymethyl lysine; CE: collision energy; CAV: collision cell accelerator voltage.

## Data Availability

The data presented in this study are available in article and Supplementary Materials.

## Supplementary Materials

The following supporting information can be downloaded at: https://www.mdpi.com/article/10.3390/molecules29061304/s1, Table S1: CML content of selected Indonesian foods.
