# Supplementary material for: Liquid Chromatography with Tandem Mass Spectrometry Analysis of Carboxymethyl Lysine in Indonesian Foods
Source: Molecules. 2024 Mar 15;29(6):1304. doi: 10.3390/molecules29061304 (PMC10975859; doi:10.3390/molecules29061304)
Supplement: Supplementary file 1 [file molecules-29-01304-s001.zip › molecules-2860689-supplementary.pdf]

Liquid Chromatography with Tandem Mass Spectrometry Analysis of Carboxymethyl Lysine in Indonesian Foods †

Patricia Budihartanti Liman [1,2,*], Mulyana [3], Yenny [4] and Ratna Djuwita [5]

[1] Department of Nutrition, Faculty of Medicine, Universitas Trisakti, Jakarta 11440, Indonesia
[2] Nutrition Study Center, Faculty of Medicine, Universitas Trisakti, Jakarta 11440, Indonesia
[3] Department of Clinical Pharmacy, Faculty of Pharmacy, Padjadjaran University, Bandung 45363, Indonesia; arsenicosa10@gmail.com
[4] Department of Pharmacology and Pharmacy, Faculty of Medicine, Universitas Trisakti, Jakarta 11440, Indonesia; yennyfarmako@trisakti.ac.id
[5] Department of Epidemiology, School of Public Health, Universitas Indonesia, Depok 16424, Indonesia; djuwita257@gmail.com
* Correspondence: patricialiman@trisakti.ac.id
† Chemical compound studied in this article: Nε-carboxymethyl lysine (PubChem CID: 123800).

Table S1. CML content of selected Indonesian foods

English name	Indonesian name	mgCML / 100g edible food	mgCML / kg protein	mgCML / average portion
**Cereals**				
Coconut cakelets	Bandros	0.14	38.57	0.12
Rice vermicelli, boiled and fried	Bihun goreng	0.95	357.14	1.43
Rice vermicelli, boiled	Bihun rebus	0.21	169.35	0.24
Biscuit, chocolate sandwich, manufactured food	Biskuit coklat sanwich	0.45	60.81	0.12
Biscuit, cream crackers, manufactured food	Biskuit cream cracker	0.84	113.51	0.23
Biscuit, with coconut, manufactured food	Biskuit kelapa	0.07	9.46	0.02
Biscuit, sweet crackers	Biskuit malkist	0.78	144.44	0.21
Biscuit crackers with chocolate, manufactured food	Biskuit malkist coklat	2.77	291.58	0.58
Biscuit, milk with vanilla flavour, manufactured food	Biskuit susu rasa vanila	0.79	121.54	0.08
Wafer, with cheese cream, manufactured food	Biskuit wafer krim keju	0.01	1.00	0.00
Wafer, with chocolate flavour, manufactured food	Biskuit wafer rasa coklat	0.11	20.91	0.02
Wafer roll, with chocolate cream, manufactured food	Biskuit wafer roll dengan krim coklat	0.04	6.40	0.01
Cake, modern with brown sugar	Bolu kukus gula merah	0.06	15.96	0.03

English name	Indonesian name	mgCML / 100g edible food	mgCML / kg protein	mgCML / average portion
Rice porridge	Bubur nasi	0.20	121.95	0.54
Tapioca flour sauce	Bumbu kuning sate padang	0.15	84.27	0.48
Cakwe, snack, fried dough	Cakwe	0.20	23.61	0.03
Tapioca dough with spring roll skin, fried	Cibay	0.20	105.26	0.13
Tapioca dough, boiled	Cilok	0.24	65.22	0.17
Starch dough, fried	Cimol	5.35	7535.21	3.32
Tapioca dough, fried	Cireng	0.15	288.46	0.03
Tapioca dough, fried	Citruk	0.06	83.33	0.03
Sereal cornflakes, manufactured food	Cornflakes	1.19	159.52	0.48
Cereal snack, cripsy oat choco, manufactured food	Cripsy oat choco	1.93	483.71	0.19
Snack, traditional cake, sweet dish, manufactured food	Dodol	0.05	14.62	0.01
Doughnuts ring	Donat	0.04	6.11	0.01
Corn, boiled	Jagung rebus	0.04	11.30	0.04
Sweet snack, made from rice flour, glutinous rice flour, and brown sugar	Juadah	0.05	25.00	0.05
Crackers made from rice flour, coconut milk, fried, manufactured food	Kerupuk ladu arai pinang	0.31	46.55	0.03
Rice cake boiled in a rhombus-shaped packet of plaited young coconut leaves	Ketupat	0.23	103.60	0.41
Cake, modern	Kue bolu	0.03	4.40	0.01
Snack, soft glutinous rice flour cake, filled with sweet grated coconut.	Kue bugis	0.67	168.34	0.19
Peanut cookies	Kue kering kacang tanah	0.80	99.75	0.11
Layered soft rice flour pudding	Kue lapis	0.26	247.62	0.13
Thousand layer cake	Kue lapis legit	0.14	31.53	0.17
Snack made from rice flour, coconut milk and sugar, filled with slices of banana, steamed in banana leaves	Kue Nagasari / kue pisang	0.05	19.23	0.03
Snack, traditional cake made from flour and coconut milk	Kue talam	0.01	17.24	0.01
Rice pasta, fried	Kwetiaw goreng	0.32	69.87	0.39

English name	Indonesian name	mgCML / 100g edible food	mgCML / kg protein	mgCML / average portion
Sticky rice dumpling with coconut milk in bamboo tubes, grilled for 120 minutes	Lemang	0.12	32.79	0.11
Sticky rice dumpling with banana	Lepat pisang	0.16	125.00	0.10
Sticky rice dumpling, with grated coconut, wrapped with coconut leaves	Leupeut	0.36	132.35	0.14
Plain rice rolls, boiled	Lontong	0.38	263.89	0.37
Rice rolls filled with carrot, boiled	Lontong isi wortel	0.31	120.16	0.19
Snacks, from corn with cheeky flavor, manufactured food	Makanan ringan ektrudat kemasan, terbuat dari jagung dengan rasa kaldu ayam	0.77	77.00	0.08
Snacks, made of wheat flour, corn, and potatoes, potato barbeque flavor, manufactured food	Makanan ringan kemasan rasa kentang	0.03	6.00	0.01
Macaroni, boiled	Makaroni, rebus	0.15	28.25	0.08
Pancakes with sweet filling	Martabak manis	0.58	133.64	0.40
Noodle, boiled	Mi basah	2.26	474.79	5.29
Instant noodles, fried noodles, brand A, manufactured food product from Indonesia	Mi instan goreng brand A	0.02	2.00	0.03
Instant noodles, fried noodles, brand B, manufactured food product from Indonesia	Mi instan goreng brand B	0.14	14.89	0.23
Instant noodles, fried noodles, brand C, manufactured food product from Indonesia	Mi instan goreng, rasa ayam kremes, brand C	0.03	3.00	0.07
Instant noodles, chicken flavor, brand A, manufactured food product from Indonesia	Mi instan, rasa ayam bawang, brand A	0.02	2.50	0.03
Instant noodles, chicken flavor, brand B, manufactured food product from Indonesia	Mi instan, rasa ayam bawang, brand B	0.24	23.76	0.32
Instant noodles, chicken flavor, brand D, manufactured food product from Indonesia	Mi instan, supermi, rasa ayam bawang, brand D	0.30	42.86	0.48
Instant noodle	Mi instant	0.13	14.31	0.22
Noodle, yellow, boiled	Mi kuning rebus	0.23	42.47	0.17
Egg noodles	Mi telur	0.19	53.98	0.07
Cereal drink, vanilla flavour, manufactured food	Minuman sereal, rasa vanilla	1.86	531.43	0.54
Fried rice with corn	Nasi goreng jagung	1.35	356.20	1.47
Fried rice with egg	Nasi goreng telur	0.84	175.73	0.84

English name	Indonesian name	mgCML / 100g edible food	mgCML / kg protein	mgCML / average portion
Turmeric rice, steamed	Nasi kuning	1.58	395.99	1.58
Spiced rice, steamed	Nasi liwet	0.06	18.07	0.12
Rice, white, cooked	Nasi putih	0.52	171.62	0.52
White rice with stir-fry fermented soybean	Nasi tutug oncom	0.43	107.77	0.43
Tapioca crackers, grilled	Opak bakar	0.07	38.46	0.01
Corn-and-wheat flour, fried	Perkedel jagung	0.10	18.25	0.04
Pancake, made from rice flour, coconut milk, and sugar	Pinukuik	0.42	140.00	0.14
Snack from rice, fried	Rengginang	0.01	1.62	0.00
Bread, filled with chocolate	Roti coklat	0.10	13.26	0.07
Bread, filled with coconut	Roti kelapa	0.06	9.79	0.04
White bread	Roti tawar	0.52	76.47	0.60
Savory and spicy dish	Seblak	0.19	71.16	0.37
Cereal chocolate balls	Sereal gandum coklat	0.88	110.00	0.18
Fermented sticky [glutinous] black rice	Tape ketan hitam	2.49	331.12	0.57
**Starchy food**				
Potato, stir-fry with spice mixture	Kentang goreng balado	0.30	81.97	0.15
potato, boiled	Kentang rebus	0.13	151.16	0.13
Chips, cassava, home made	Keripik singkong, produk rumahan	0.06	31.25	0.01
Chips, from cassava flour, fried	Kerupuk aci	0.10	103.31	0.04
Crackers made from flour, fried	Kerupuk merah padang	0.11	220.00	0.01
Potato chips, manufactured food	Makanan ringan kemasan, keripik kentang	0.82	82.00	0.16
Cracker, cassava, fried, home made	Opak beca, produk rumahan	0.06	66.67	0.02
Snack, grated casava filled with sweet grated coconut and brown sugar, steamed	Papais Sampeu	0.10	75.76	0.08
Cassava, white, boiled	Singkong rebus	0.15	100.00	0.16
Fermented cassava	Tape singkong	0.10	208.33	0.18
Yam, fried with flour	Ubi goreng tepung	0.43	169.29	0.58
Yam, boiled	Ubi rebus	0.16	74.77	0.19
**Legumes**				
Dumpling, chicken and tofu, steamed	Bakso tahu, rebus	0.14	10.46	0.15
Peanut sauce	Bumbu kacang	0.18	64.98	0.28

English name	Indonesian name	mgCML / 100g edible food	mgCML / kg protein	mgCML / average portion
Tofu, filled with vegetables, fried	Gehu / tahu isi	0.88	61.93	0.45
Stinky beans, boiled and fried with spice mixture	Jengkol balado	0.17	31.08	0.09
Peanut coated with tapioca flour, fried, manufactured food	Kacang bersalut, rasa original	4.30	383.93	0.60
Cowpea, fried	kacang tolo/tunggak, goreng	1.09	146.51	0.22
Soybean, fried	kacang kedelai, goreng	0.88	26.62	0.22
Peanut, fried	Kacang tanah, goreng	0.06	2.39	0.04
Peanut, boiled	Kacang tanah, rebus	0.40	28.17	0.11
Cowpea, boiled	Kacang tolo/tunggak, rebus	1.42	249.12	0.21
Soy, ketchup	Kecap manis	0.61	27.88	0.37
Tempeh, lightly fried battered	Mendoan tempe/cipe	0.63	76.64	0.37
Fermented peanut press cake, stir-fry	Oncom, tumis	1.69	477.40	0.68
Tofu, steamed with spices, and wrapped in banana leaf	Pepes tahu	1.35	101.96	1.13
Rice flour with peanut, fried	Rempeyek kacang	0.43	24.57	0.12
Tofu, fried	Tahu goreng	1.13	78.15	0.90
Tofu, fried and stir-fry with spice mixture	Tahu goreng balado	0.18	10.34	0.06
Tofu filled with rice vermicelli, fried with flour	Tahu isi bihun goreng	0.02	1.69	0.01
Tofu, boiled with soy ketchup	Tahu semur	1.81	136.71	1.45
Tempeh, fried	Tempe goreng	0.67	31.17	0.18
**Meat and poultry**				
Chicken gizzard, fried for 4 minutes	Ampela ayam goreng	0.27	7.63	0.03
Chicken, meat, thigh, grilled	Ayam bakar paha (ayam pejantan)	1.24	52.41	0.67
Chicken, meat, breast, fried	Ayam dada goreng	0.99	24.00	0.43
Chicken, meat, breast, boiled	Ayam dada rebus	0.15	6.35	0.06
Chicken, thigh, fried	Ayam paha goreng	0.53	14.51	0.20
Chicken, meat, breast, grilled	Ayam, dada, bakar (ayam pejantan)	1.12	45.42	0.69
Meatballs, beef and innards, boiled	Bakso Daging Sapi Isi Jeroan, rebus	0.27	48.47	0.46
Meat balls, fried	Bakso goreng	0.04	74.07	0.01
Meat balls, boiled	Bakso polos, daging sapi, rebus	1.50	171.53	2.55
Meatballs, beef, boiled	Bakso Urat, rebus	2.00	168.07	3.40

English name	Indonesian name	mgCML / 100g edible food	mgCML / kg protein	mgCML / average portion
Chicken, shredded, boiled	Daging ayam suir, rebus	0.16	4.51	0.06
Beef soup, boiled	Daging sapi rebus, soup	0.07	4.32	0.03
Chicken, meat and skin, breast, fried with flour	Fried chicken, dada	1.81	69.14	1.67
Chicken, meat and skin, wing, fried with flour	Fried chicken, sayap	2.81	229.58	1.66
Chicken, meat, breast, boiled with coconut milk	Gulai ayam, dada	4.41	167.05	2.78
Chicken, meat, drumstick, boiled with coconut milk	Gulai ayam, paha	1.62	64.13	0.58
Meat, beef, thigh, Gulai, boiled for 90 minutes with coconut milk	Gulai daging sapi	1.12	29.24	0.26
Chicken, liver, fried for 4 minutes	Hati ayam, goreng	0.32	9.31	0.10
Meat, beef, thigh, Kalio, boiled for 140 minutes with coconut milk	Kalio daging sapi	1.21	34.53	0.23
Cow skin crackers, fried	Kerupuk kulit sapi	0.87	12.58	0.26
Snack from dough filled with mix of egg, meat, onion	Martabak mesir	0.34	43.37	0.25
Nuggets, chicken, fried	Nugget ayam, goreng	0.28	17.57	0.32
Chicken, breast, streamed with spices, and wrapped with banana leaf	Pepes ayam	0.56	23.58	0.60
Meat, beef, thigh, Rendang, boiled for 190 minutes for with coconut milk	Rendang daging sapi	1.72	47.82	0.65
**Fish, shellfish, and shrimps**				
Fish, three spot gourami, salted, fried for 3.5 minutes	Asin ikan sepat, goreng	0.61	13.69	0.09
Fish, jambal roti, salted, dried, fried for 3.5 minutes	Asin jambal roti, goreng	0.83	17.15	0.12
Milkfish, processed, fried	Bandeng pindang, goreng	0.90	33.59	0.46
Fish dumpling, fried	Batagor pangsit	0.14	16.26	0.12
Mackerel, fried	Ikan kembung, goreng	0.78	24.84	0.42
Ponyfish, fried	Ikan maco, goreng	0.02	0.37	0.01
Carp, fried for 10 minutes	Ikan mas, goreng	0.64	27.79	0.24
Tilapia, fried	Ikan mujahir, goreng	0.17	5.63	0.15
Nile tilapia, boiled	Ikan nila asam padeh	2.03	106.28	0.99
Pinang-pinang fish, fried and stir-fry with spicy mixture	Ikan pinang-pinang balado	0.68	27.20	0.20
Scad, fried	Ikan sarai, goreng	1.23	33.09	0.63
Tarontong fish, grilled	Ikan tarontong, bakar	0.05	1.98	0.02

English name	Indonesian name	mgCML / 100g edible food	mgCML / kg protein	mgCML / average portion
Anchovy, dried, salted, fried with spice mixture	Ikan teri balado	0.11	2.78	0.02
Anchovy, dried, salted, fried	Ikan teri, goreng	1.55	30.14	0.40
Mackerel tuna, boiled with coconut milk	Ikan tongkol, gulai	0.12	4.78	0.07
Crackers made from flour and salted fish, fried, manufactured food	Kerupuk rakik Ikan kemasan, goreng	5.19	281.45	2.34
Bilis fish, grilled	Palai Ikan bilis	0.18	7.83	0.04
Bilis fish with coconut and cassava leaf, wrapped with banana leaf, grilled	Palai ikan bilis dengan kelapa dan daun singkong	0.20	20.94	0.20
Sasau fish, boiled	Pangek ikan sasau	0.88	56.41	0.84
Fermented fish, fried	Peda goreng	1.19	50.25	0.13
Fermented fish, grilled	Pepes peda	0.53	28.80	0.12
Fish finger, fried	Sala ikan, goreng	0.41	151.85	0.09
Dumpling, fish, steamed	Siomay ikan	0.48	79.73	0.36
Shrimp paste, fermented	Terasi udang	0.31	10.11	0.01
Shrimp stir-fry with sour and sweet sauce	Udang goreng asam manis	0.05	2.18	0.01
**Eggs**				
Egg yolk, chicken, boiled	Kuning telur ayam, rebus	0.60	40.54	0.09
Egg yolk, quail, boiled	Kuning telur puyuh, rebus	0.82	62.12	0.16
Egg white, chicken, boiled	Putih telur ayam, rebus	0.32	25.00	0.14
Egg white, quail, boiled	Putih telur puyuh, rebus	0.11	6.60	0.04
Egg, chicken, boiled and stir-fry with spice mixture	Telur ayam balado	0.99	70.71	0.53
Egg, chicken, fried	Telur ayam goreng	0.84	55.63	0.42
Egg, chicken, boiled	Telur ayam rebus	0.28	20.59	0.16
Omelet	Telur ayam, dadar	0.51	34.42	0.32
Egg, duck, salty, boiled	Telur bebek asin, rebus	0.10	7.35	0.07
Egg, carp, freshwater, fried for 10 minutes	Telur ikan mas goreng	2.10	108.81	0.32
Egg, quail, boiled	Telur puyuh rebus	0.65	43.62	0.07
Egg, boiled with soy kechup	Telur Semur	0.04	3.17	0.02
**Milk product, chocolate, and coffee**				
Milk chocolate-coated wafers, manufactured food	Coklat dengan susu dan wafer	0.95	161.02	0.16

English name	Indonesian name	mgCML / 100g edible food	mgCML / kg protein	mgCML / average portion
Dairy milk chocolate (chocolate 28%)	Coklat susu	0.79	106.76	0.21
Coffee	Kopi bubuk	0.13	7.82	0.01
3 in 1 instant coffee (sugar, non diary creamer, and 15% instant coffee)	Kopi instant 3 in 1	0.14	40.23	0.03
Powdered milk	Susu bubuk	0.25	10.42	0.03
Sweet condensed milk	Susu kental manis putih	2.79	1116.00	1.12
Ultra-high-temperature pasteurized milk, chocolate flavour	Susu UHT rasa coklat	0.23	115.00	0.46
**Vegetables**				
Coconut and cassava leaf grilled	Kelapa Parut, Daun singkong	0.20	38.68	0.16
Leunca peanut, stir-fry	Leunca, tumis	0.33	300.00	0.07
**Processed fruit**				
Banana chips, fried, home made	Keripik pisang, produk rumahan	0.07	30.17	0.01
Banana, fried with flour and chocolate	Pisang aroma	0.12	13.90	0.03
Banana, fried with flour	Pisang goreng	0.13	47.45	0.10
Banana, pastry puff	Pisang molen	1.31	642.16	1.55
**Mixed food dishes**				
Dumpling, fish and tofu, fried	Batagor pangsit dan tahu	0.14	13.91	0.23
Fermented peanut press cake and leunca peanut, stir-fry	Bolokotok leunca	0.22	74.35	0.18
Rice porridge, with chicken boiled, fried cakwe, fried soy bean, and chips	Bubur ayam	0.21	57.63	0.68
Coffee with sweet condensed milk	Kopi susu dengan susu kental manis	0.05	20.00	0.10
Rice dumpling in palm leaf pouch, boiled with fried tofu and peanut sauce	Kupat tahu	1.09	219.58	5.46
Noodle, with chicken	Mi ayam	1.96	231.26	4.51
Turmeric rice, steamed with fried salted anchovy, sauteed fried tempeh with soy kechup, and sliced omelette	Nasi kuning	1.26	98.34	4.17
